# Establishment of Reverse Transcription Polymerase Chain Reaction Method for Quantification of Residual Host Cell RNA in Recombinant Plasmid Drugs

**DOI:** 10.3390/molecules31203670

**Published:** 2026-10-14

**Authors:** Hua Bi, Bo Zhang, Xinchang Shi, Xiang Li, Dening Pei, Shanshan Ma, Huili Hou, Ying Guo, Youxue Ding, Xi Qin, Chenggang Liang

**Affiliations:** 1 National Institutes for Drug Control, Beijing 100050, China; bihua@nidc.org.cn (H.B.); shixc@nidc.org.cn (X.S.); lix@nidc.org.cn (X.L.); peidening@nidc.org.cn (D.P.); guoying@nidc.org.cn (Y.G.); dingyouxue@nidc.org.cn (Y.D.); 2 Beijing Northland Biotechnology Co., Ltd., Beijing 100085, China; zhangbo@northland-bio.com (B.Z.); mashanshan@northland-bio.com (S.M.); houhuili@northland-bio.com (H.H.)

**Keywords:** recombinant plasmid, residual host cell RNA, quantitative reverse transcription polymerase chain reaction, quality control

## Abstract

The presence of residual host cell RNA in recombinant plasmid drugs poses potential safety concerns and is subject to strict regulatory limits. Therefore, reliable quantification methods are essential for quality control. This study aims to establish and validate a reverse transcription-quantitative polymerase chain reaction (RT-qPCR) method for the quantitative detection of residual *Escherichia coli* host cell RNA in recombinant plasmid drugs. The DNA digestion system was optimized, and three RNA reference materials from different sources (kit-supplied RNA, commercial Thermo Fisher RNA, and self-extracted RNA) were compared. The results for 2-, 4-, and 8-fold diluted samples were comparable. The three reference materials showed comparable electrophoretic band patterns and amplification curves, and they also yielded comparable RNA quantification results for the same samples. After systematic methodological validation of the established RT-qPCR method, it was applied to seven batches of recombinant plasmid drug substances and three batches of in-process intermediates. Notably, residual host cell RNA levels in all tested batches were below 0.2 μg/mg DNA, complying with current national and regional regulatory requirements.

## 1. Introduction

Recombinant plasmids are circular DNA molecules constructed by inserting exogenous genes or nucleic acid sequences into modified plasmid vectors using molecular biology techniques [1]. Plasmids are naturally occurring double-stranded, circular, extrachromosomal DNA molecules capable of autonomous replication in bacteria [2]. In genetic engineering, plasmids are modified to retain essential elements such as replication origins, multiple cloning sites, and selectable markers, enabling their use as vectors for the delivery and expression of specific genes. As one of the core tools for gene delivery, recombinant plasmids hold significant value in both therapeutic applications and basic research. They serve as research tools to facilitate gene transfer and functional exchange in microbial genetics. More importantly, they are utilized as non-viral vectors in gene therapy, including naked plasmid DNA drugs and DNA vaccines. The construction, preparation, and quality control strategies for these gene therapy products must comprehensively address considerations of efficacy, safety, and regulatory compliance [3,4,5,6].

For gene therapy plasmid drugs, the production process typically relies on the amplification of host cells, such as *Escherichia coli*. During this process, host cell-derived impurities—including RNA, DNA, and proteins—may be introduced into the final product. These impurities can not only compromise product purity and stability but also trigger unwanted immune responses, thereby posing significant risks to the safety, efficacy, and consistency of the final biological product [7,8,9]. Consequently, the detection of residual host cell RNA has become a critical quality attribute in the quality control of recombinant plasmid-based biologics.

Major regulatory agencies worldwide have established stringent requirements for the control of residual host cell RNA. For instance, the Technical Guidelines for Pharmaceutical Research and Evaluation of In Vivo Gene Therapy Products (Trial) issued by the Center for Drug Evaluation (CDE) of China’s National Medical Products Administration recommends that residual host cell RNA be included as a quality specification for plasmid DNA [10]. The United States Pharmacopeia (USP–NF) General Chapter <1047> Gene Therapy Products specifies that residual RNA should be less than 0.2 μg/mg DNA [11]. Similarly, the European Medicines Agency (EMA) states in its Guideline on the Quality, Non-clinical and Clinical Aspects of Gene Therapy Medicinal Products that host cell residual RNA should be considered a process-related impurity, with levels controlled through the establishment of quality specifications or process validation to ensure product safety and quality [12].

For the detection of residual host cell DNA and proteins, well-established methods and acceptance limits are clearly defined in major pharmacopoeias and regulatory guidelines. In contrast, the detection of residual host cell RNA has primarily relied on techniques such as agarose gel electrophoresis and high-performance liquid chromatography (HPLC) [11]. Reverse transcription quantitative polymerase chain reaction (RT-qPCR) is a technique that reverse transcribes RNA into complementary DNA (cDNA) followed by real-time fluorescent quantitative PCR amplification. Owing to its high sensitivity, excellent specificity, and broad dynamic range, RT-qPCR has become a key analytical tool in drug development, manufacturing, and quality control [13,14,15,16]. The aim of this study was to establish an RT-qPCR quantitative method with high specificity, sensitivity, and repeatability for the detection of *E. coli* residual host cell RNA in recombinant plasmid products, thereby providing a foundation for the quality control of recombinant plasmid-based biologics.

## 2. Materials and Methods

### 2.1. Materials and Reagents

*E. coli* total RNA reference material:

*E. coli* total RNA (Thermo Fisher Scientific (Waltham, MA, USA), catalog number: AM7940).

*E. coli* RNA quantitative reference (Junyan Biotechnology Co., Ltd. (Shanxi, China), catalog number: RC0311B-100).

Self-extracted RNA (prepared in the laboratory using *E. coli* DH5α and a total RNA extraction kit).

Reagents:

Total RNA extraction kit (Shanghai Promega Biological Products Co., Ltd. (Shanghai, China), catalog number: LS1040), LB Broth (without sugar) (Guangdong Huankai Microbial Sci.&Tech. Co., Ltd., (Guangzhou, China), catalog number: 028324), Agarose (Biowest (Nuaillé, France), distributed by Shanghai Baygene Biotechnology Co., Ltd. (Shanghai, China), catalog number: BY-R0100), 50 × TAE Buffer (Jiangsu Cowin Biotech Co., Ltd. (Taizhou, China), catalog number: CW0663S), GelRed Nucleic Acid Gel Stain (BIOTIUM (Fremont, CA, USA), catalog number: 41003), 6 × Loading Buffer (Takara Bio Inc. (Kusatsu, Shiga, Japan), catalog number: 9156), BS15000 DNA Marker (Bosun Life (Beijing, China), catalog number: BS1103), DNase I, RNase-free (Nanjing Vazyme Biotechnology Co., Ltd. (Nanjing, China), catalog number: EN401-01), AccurSTART II U+ One Step RT-qPCR Probe Kit (containing 5 × One Step U+ Mix, One Step U+ Enzyme Mix, and 50 × ROX Reference Dye) (Nanjing Vazyme Biotechnology Co., Ltd., catalog number: Q233-02), DNase/RNase-free deionized water (Tiangen Biochemical Technology Co., Ltd. (Beijing, China), catalog number: 1109285). Primers and probe were synthesized by Sangon Biotech Co., Ltd. (Shanghai, China).

Primers and probe:

Forward primer: 5′-CCTTACGACCAGGGCTACACA-3′.

Reverse primer: 5′-CTCGCGAGGTCGCTTCTC-3′.

Probe: 5′FAM-CGTGCTACAATGGCGCATACA-BHQ13′.

The primer and probe sequences were adopted from USP-NF <509> Residual DNA Testing [17]. They target the 16S rRNA gene of *E. coli*, with an amplicon size of 62 bp.

### 2.2. Instruments

Gel imaging system (GelDoc Go, Bio-Rad Laboratories, Inc., Hercules, CA, USA), electrophoresis power supply (DYY-6C, Beijing Liuyi Biotechnology Co., Ltd., Beijing, China), real-time fluorescence quantitative PCR instrument (QuantStudio 5, Thermo Fisher Scientific, Waltham, MA, USA), microvolume spectrophotometer (NanoDrop One, Thermo Fisher Scientific), biological safety cabinet (BSC-1304IIA2, Suzhou Antai Airtech Co., Ltd., Suzhou, China).

### 2.3. Recombinant Plasmid Drugs

The recombinant plasmid drugs used in this study were provided by Beijing Northland Biotechnology Co., Ltd. (Beijing, China), under the product name Recombinant Human Hepatocyte Growth Factor Naked Plasmid Injection. The batch numbers of the drug substance were 01JC202311003–005 and NLJC202412001–004. Process intermediates were selected from the three-step chromatography pools corresponding to batches NLJC202412001–003.

### 2.4. Establishing the Method

#### 2.4.1. Standard Solution Preparation

The RNA reference substance was serially diluted with RNA diluent to obtain final concentrations of 200, 20, 2, 0.2, 0.02, and 0.002 pg/μL.

#### 2.4.2. Study on DNA Digestion System

Prior to RT-qPCR, DNA was removed from the test samples. The digestion mixture was prepared in RNase-free centrifuge tubes according to Table 1, with a total volume of 40 μL. The test sample volumes were 20, 10, and 5 μL, corresponding to dilution factors of 2-, 4-, and 8-fold, respectively.

The above solution was incubated in a metal bath at 37 °C for 15 min. After removal, 4 μL of 50 mmol/L EDTA solution was added to terminate the reaction, and the mixture was mixed thoroughly to serve as the RNA template.

#### 2.4.3. Comparison of RNA Reference Materials

- Preparation of self-extracted *E. coli* DH5α RNA

A 1 mL aliquot of *E. coli* DH5α culture was inoculated into 50 mL of LB medium and incubated overnight at 37 °C with shaking at 210 rpm. RNA was extracted from 1 mL of the bacterial culture using the total RNA extraction kit according to the manufacturer’s instructions, and the RNA concentration and purity were determined using a micro-ultraviolet spectrophotometer by measuring the absorbance at 260 nm and the A260/A280 ratio, respectively.

- Agarose gel electrophoresis analysis of RNA reference materials

The self-extracted RNA, *E. coli* RNA quantitative reference, and *E. coli* total RNA were subjected to agarose gel electrophoresis. Briefly, a 0.8% (*w*/*v*) agarose gel was prepared in 1× TAE buffer, with 5 μL of GelRed Nucleic Acid Gel Stain added per 50 mL of gel solution. This gel solution was then poured into a gel-casting mold and allowed to solidify at room temperature for 30 min.

The three RNA reference samples were diluted with nuclease-free water to a final concentration of 0.1 μg/μL. For sample preparation, 10 μL of each diluted sample was mixed with 2 μL of 6× Loading Buffer to obtain the test solutions. Subsequently, 6 μL of BS15000 DNA Marker and each prepared test solution were loaded into separate wells of the agarose gel. Electrophoresis was performed at a constant voltage of 140 V for 35 min. Upon completion of electrophoresis, the gel was imaged and analyzed using a gel imaging system (software version 3.0.0.07) under UV trans-illumination with the GelRed detection program.

- RT-qPCR comparison of RNA reference materials

After verification via agarose gel electrophoresis, the three RNA reference materials were subjected to RT-qPCR to obtain their respective amplification curves. Meanwhile, the identical three batches of test samples were assayed using each of the above reference materials, and the detection values obtained with different reference materials were compared.

#### 2.4.4. RT-qPCR Reaction

The number of reaction wells was determined based on the number of points in the standard curve and the number of test samples. RNA diluent was used as a no-template control (NTC). The single-well RT-qPCR reaction system is shown in Table 2. The reaction conditions were as follows: reverse transcription at 55 °C for 15 min; pre-denaturation at 95 °C for 30 s; followed by 45 cycles of denaturation at 95 °C for 10 s and annealing at 60 °C for 34 s. The total reaction volume was 20 μL. FAM was used as the reporter dye and ROX as the reference dye, and the quencher option was set to none.

### 2.5. Method Validation

#### 2.5.1. Specificity

The sample buffer and test samples were used as templates for RT-qPCR detection to assess assay specificity.

#### 2.5.2. Range

- Linearity

A standard curve was constructed by plotting Ct values against the log_10_ (RNA concentration), followed by linear regression analysis to determine the coefficient of determination (R^2^) and amplification efficiency.

- Limit of Quantitation

The lowest concentration of the RNA reference (0.002 pg/μL) was tested six times using the established RT-qPCR method, and the relative standard deviation (RSD) was calculated.

#### 2.5.3. Accuracy

Samples spiked with high, medium, and low concentrations of the RNA reference were prepared by adding 4 μL of RNA reference at concentrations of 200, 20, and 0.2 pg/μL, respectively. Each concentration was tested in triplicate. Unspiked samples were also analyzed. The spike recovery for each concentration level was calculated using the following equation:

$$\mathrm{Spike}\;\mathrm{recovery}\;(\%)\;=\;\frac{D_{\mathrm{ss}}\times V_{\mathrm{ss}}-D_{s}\times V_{s}}{C_{\mathrm{th}}\times V_{a}}\;\times \;100$$

where D_ss_ is the detection value of the spiked sample, V_ss_ is the total volume of the spiked sample, D_s_ is the detection value of the sample, V_s_ is the total volume of the sample, C_th_ is the theoretical spiked concentration, and V_a_ is the spiked volume.

#### 2.5.4. Precision

- Repeatability

Six replicates of the test samples and six replicates of the spiked samples were prepared and analyzed by RT-qPCR. The relative standard deviation (RSD) was calculated for each group.

- Intermediate Precision

Two analysts independently prepared six replicates of the test samples and six replicates of the spiked samples on different days, and the samples were analyzed by RT-qPCR. The RSD was calculated for each group.

### 2.6. Preliminary Application of the Method

The established method was used to quantitatively detect residual host cell RNA in seven batches of recombinant plasmid drug substance samples and three batches of process intermediate samples, and the results were evaluated.

## 3. Results

### 3.1. Study on DNA Digestion System

Test samples were subjected to DNA digestion after dilution factors of 2, 4, and 8. Comparable RNA contents were observed across the three dilution conditions, with spike recoveries ranging from 111.46% to 119.91% (Table 3), which meets the acceptable range of 70–130%. Based on these results, the 2-fold dilution system was selected for the DNA digestion step in the established method.

### 3.2. Comparison of RNA Reference Materials

The concentration of the self-extracted RNA was 106.0 ng/μL, with an A260/A280 ratio of 2.05. Agarose gel electrophoresis (1%) revealed that the self-extracted RNA was of good purity, and the bands of the self-extracted RNA, *E. coli* RNA quantitative reference, and *E. coli* total RNA were comparable (Figure 1). RT-qPCR results showed that the amplification curves of the three RNA reference materials were similar, and the calculated RNA contents were also comparable across the different amplification curves (Figure 2 and Figure 3 and Table 4). In summary, all three RNA reference materials were suitable for this study. Based on a comprehensive consideration of accessibility and cost, *E. coli* total RNA was used as the RNA reference material in subsequent validation experiments.

### 3.3. Method Validation

#### 3.3.1. Specificity

The detected value for the sample buffer was 0 pg/μL, while that for the test sample was 0.017 pg/μL, with a spike recovery of 96.9%. These results indicate that the method exhibits good specificity.

#### 3.3.2. Range

- Linearity

Within the RNA concentration range of 0.002 pg/μL to 200 pg/μL, the Ct value was linearly correlated with the log_10_ (RNA concentration). The coefficient of determination (R^2^) was 1.000, and the amplification efficiency was 97.327%. No signal was detected for the negative control (NCS) or the no-template control (NTC) (Figure 4).

- Limit of Quantitation

The relative standard deviation (RSD) for the lowest concentration of the reference substance (0.002 pg/μL) was 3.9%, and the spike recovery ranged from 70.0% to 130.0%. The limit of quantitation (LOQ) of the method was determined to be 0.002 pg/μL.

#### 3.3.3. Accuracy

The spike recoveries for the high-, medium-, and low-concentration spiked samples were 123.2%, 101.3%, and 96.1%, respectively, all falling within the acceptable range of 70% to 130% (Table 5). These results indicate that the method exhibits good accuracy.

#### 3.3.4. Precision

- Repeatability

The relative standard deviations (RSDs) for the six replicate test samples and six replicate spiked samples were 11.5% and 7.3%, respectively, both below 30% (Table 6). These results indicate that the method exhibits good repeatability.

- Intermediate Precision

The relative standard deviations (RSDs) for the 12 test samples and 12 spiked samples were 9.5% and 10.8%, respectively, both below 30% (Table 7). These results indicate that the method exhibits good intermediate precision.

### 3.4. Preliminary Application of the Method

Figure 5 depicts the stepwise clearance profiles of residual host cell RNA throughout the purification workflow, covering the first, second, and third chromatography pools as well as the final drug substance from three independent batches. All three batches exhibited highly consistent downward trends, demonstrating favorable batch-to-batch reproducibility of the impurity removal process. The detailed residual host cell RNA levels of seven batches of recombinant plasmid drug substance and three batches of process intermediates are summarized in Table 8. Notably, the residual RNA content in the first chromatography pool, though relatively higher than in subsequent steps, remained below 0.2 μg/mg DNA. The RNA levels decreased significantly with further purification steps, verifying that the established manufacturing process can effectively eliminate residual host cell RNA and ensure the quality of the final product.

## 4. Discussion

In this study, an RT-qPCR method for the detection of residual host cell RNA in recombinant plasmids was successfully established and systematically validated, confirming its scientific validity and reliability. In the optimization of the DNA digestion system, the test samples showed effective removal of DNA interference across dilution factors ranging from 2- to 8-fold, with stable spike recoveries; a 2-fold dilution was ultimately selected as the standard pretreatment condition, which ensured the removal of DNA while maximally retaining the RNA of interest. Regarding the selection of reference materials, no international or national standard for *E. coli* residual RNA is currently available. In this study, three sources of *E. coli* RNA reference materials—kit-matched, commercially procured, and laboratory-prepared—were compared. No significant differences were observed in electrophoretic band patterns or amplification curves, and the test results across three batches of samples were comparable, indicating that the method exhibits good compatibility with reference materials from different sources. This finding may serve as a useful reference for other researchers during reference material selection in method development. The established method was subsequently applied to the detection of drug substance and in-process samples. The results showed that although a certain level of RNA residue was present in the process intermediates, the residual host cell RNA in the final drug substance was far below the quality specification of 0.2 μg/mg DNA following subsequent purification steps, indicating that the production process effectively removes RNA and meets product quality requirements. Furthermore, the accuracy and reliability of the method were further corroborated, providing a solid foundation for technology transfer and broader application.

In this study, the primer and probe sequences recommended in USP-NF <509> Residual DNA Testing [17] were preferentially adopted rather than designing them in-house. This approach not only improves method compliance and sequence traceability but also leverages the existing application basis of the pharmacopeial primers, thereby reducing the risks of nonspecific amplification, primer dimers, and poor amplification efficiency that may arise from in-house design. Although this primer/probe set was originally designed for host cell DNA detection, Primer-BLAST alignment for primer specificity verification revealed that it targets a conserved region of the *E. coli* 16S rRNA gene, with an amplicon length of only 62 bp. Given the high abundance of 16S rRNA in host bacteria, its transcript, after reverse transcription, is highly matched to the primer/probe sequences and can therefore still be efficiently amplified in the RT-qPCR system. The DNA digestion step prior to amplification simultaneously removes plasmid and host cell DNA, ensuring that the amplification signal originates from residual host cell RNA rather than DNA.

Compared with agarose gel electrophoresis, RT-qPCR effectively distinguishes host RNA from target nucleic acids and matrix interference through the targeted recognition capability of specific primer–probe sets, achieving sensitivity at the picogram level or even lower, which is significantly superior to gel electrophoresis [18,19]. High-performance liquid chromatography (HPLC) does not require nucleic acid amplification and enables direct quantification of total RNA via chromatographic separation, yielding stable and reproducible results; however, its detection sensitivity is only at the nanogram level, which cannot meet the release specifications for biological products, though it may be suitable for quality monitoring of process intermediates [10,20]. Although high-throughput sequencing can comprehensively analyze RNA composition, fragment distribution, and potential exogenous sequences, it is associated with long detection cycles, high cost, and lower quantitative accuracy compared with RT-qPCR, making it unsuitable for routine testing [21]. Digital PCR enables absolute quantification without the need for reference standards and exhibits greater resistance to matrix interference, but its low throughput and high cost limit its use in conventional testing [22,23]. Overall, RT-qPCR offers outstanding advantages in sensitivity, specificity, throughput, and cost-effectiveness, making it a more suitable method for the detection of residual host cell RNA in drug product release testing [19,21].

Regulatory agencies also emphasize the importance of evaluating the effectiveness of each purification step in removing residual host cell RNA during drug manufacturing. The established method is also suitable for assessing the clearance of nucleic acid impurities throughout the purification process of recombinant plasmid products. In cases where the sample matrix is more complex, the method can be combined with magnetic bead-based extraction to enhance analytical sensitivity and specificity [24,25]. Given the widespread application of plasmids in biotechnological drugs, this method is also applicable to the quality control of residual RNA in recombinant plasmids used in the production of mRNA drugs, DNA vaccines, and other related products. In summary, the RT-qPCR method established in this study is sensitive, accurate, and reproducible, and can serve as a valuable reference for the quality control of recombinant plasmid-based biological products.

## Figures and Tables

**Figure 1:**
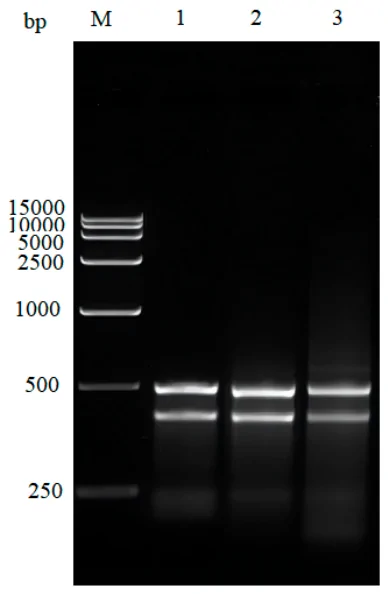
Agarose gel electrophoresis profiles of three sources of RNA reference materials. M: 15,000 DNA marker; 1: *E. coli* RNA quantitative reference; 2: *E. coli* total RNA; 3: self-extracted RNA.

**Figure 2:**
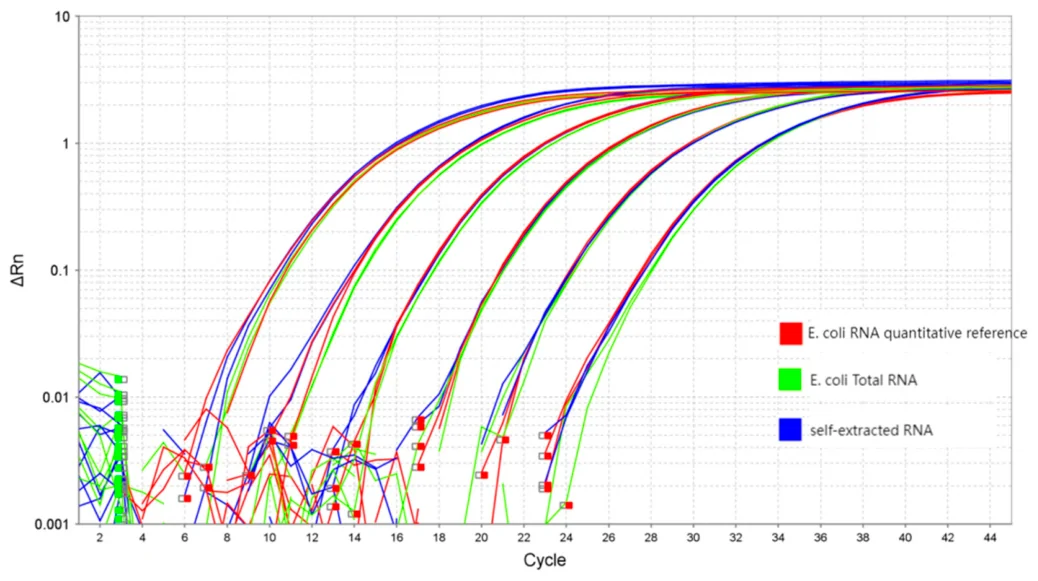
Amplification curves of three sources of RNA reference materials.

**Figure 3:**
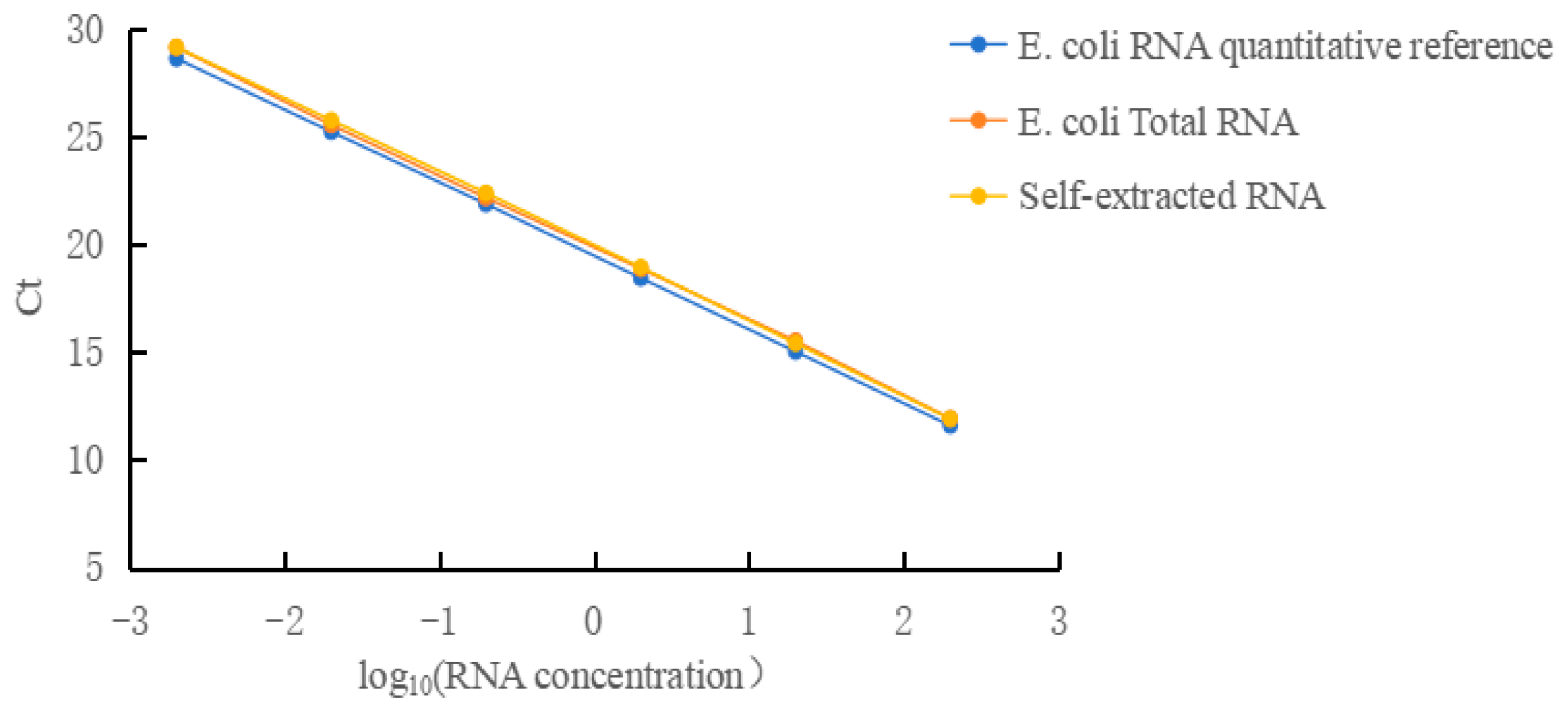
Standard curves of three sources of RNA reference materials.

**Figure 4:**
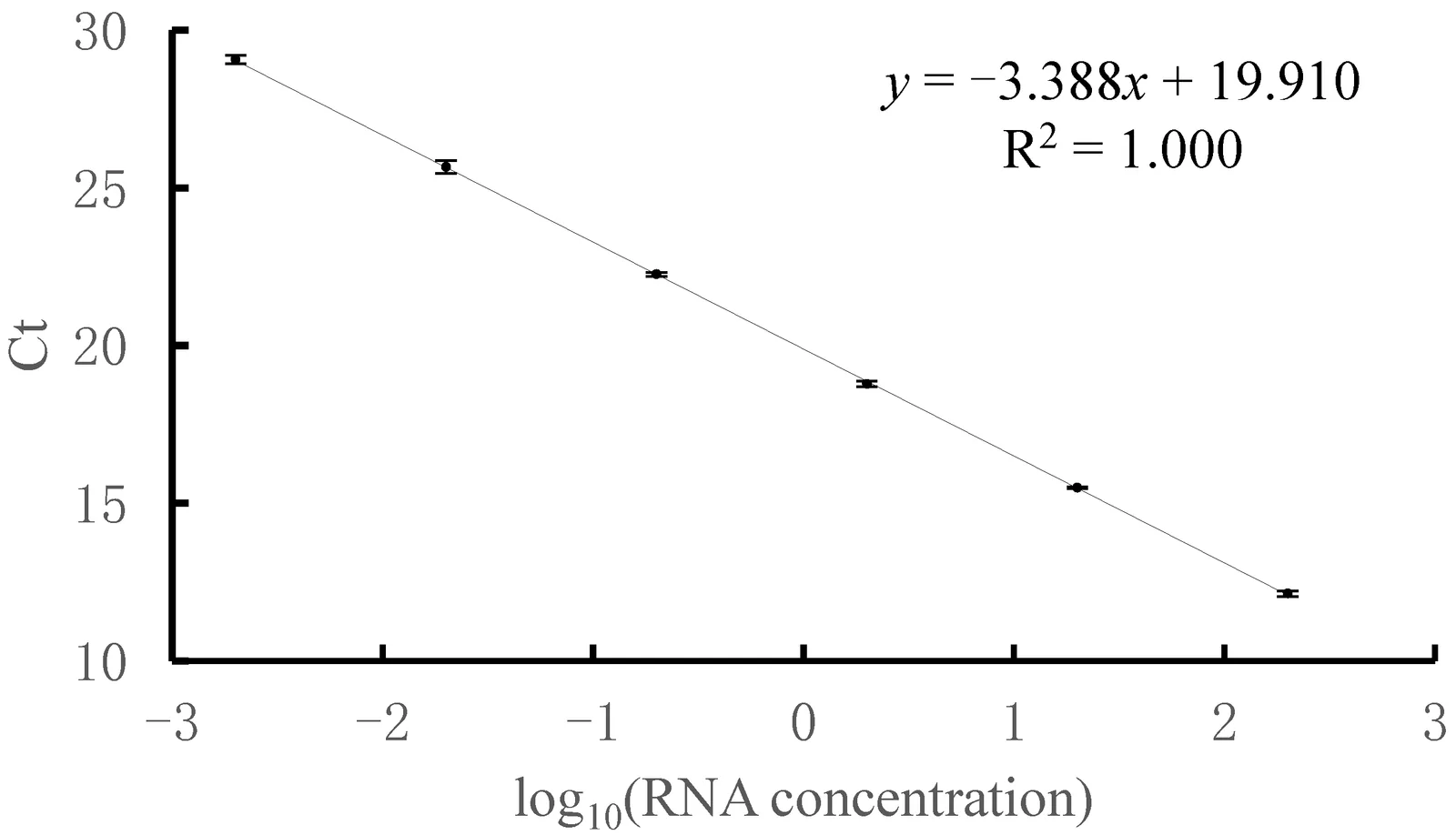
Standard curve for RT-qPCR. Error bars represent the standard deviation of triplicate measurements.

**Figure 5:**
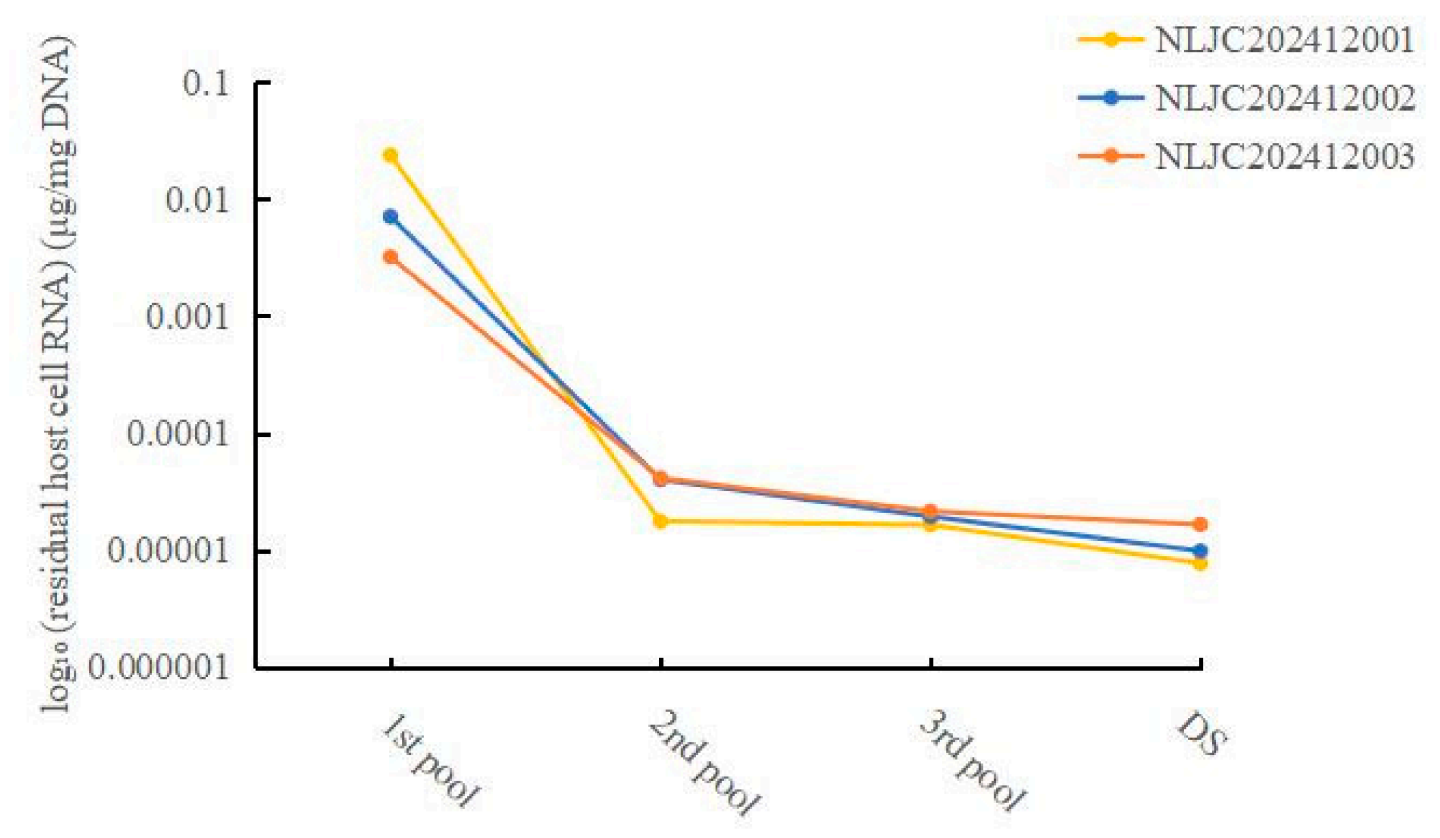
Clearance trends of residual host cell RNA during plasmid purification. Abbreviations: 1st pool, first chromatography pool; 2nd pool, second chromatography pool; 3rd pool, third chromatography pool; DS, drug substance.

**Table 1:** DNA digestion system sample preparation.

Name	Sample (μL)	Sample Dilution (μL)	Negative Control NCS0020 (μL)
DNase I, RNase-free enzyme	4	4	4
10 × Reaction Buffer	4	4	4
DNase/RNase-free deionized water	12/22/27	12/22/27	32
Test sample volume	20/10/5	20/10/5	-
RNA reference material volume	-	4	-
Total volume	40	44	40

**Table 2:** Single-well RT-qPCR reaction system.

Component	Volume (μL)
5 × One Step U+ Mix	4
One Step U+ Enzyme Mix	1
50 × ROX Reference Dye	0.4
Forward primer	0.4
Reverse primer	0.4
Probe	0.2
DNase/RNase-free deionized water	8.6
RNA template	5

**Table 3:** Results of DNA digestion system research.

Dilution Ratio	Test Value (pg/μL)	RNA Content (pg/μL)	Spike Recovery (%)
2	0.011	0.024	119.91%
4	0.005	0.022	114.76%
8	0.002	0.022	111.46%

**Table 4:** Detection results of three types of RNA reference materials.

Item		RNA Content Detection Results (pg/μL)		
		*E. coli* RNA Quantitative Reference	*E. coli* Total RNA	Self-Extracted RNA
NLJC202412001		0.018	0.018	0.018
NLJC202412002		0.026	0.035	0.031
NLJC202412003		0.044	0.053	0.044
Ct	200 pg/μL	11.586	11.922	11.901
	20 pg/μL	15.020	15.510	15.393
	2 pg/μL	18.421	18.859	18.922
	0.2 pg/μL	21.853	22.145	22.363
	0.02 pg/μL	25.204	22.521	25.734
	0.002 pg/μL	28.592	29.110	29.121
Standard curve		y = −3.400 x + 19.436	y = −3.407 x + 19.833	y = −3.445 x + 19.887
R^2^		1.000	1.000	1.000
Amplification efficiencies (%)		96.826	96.557	95.036

**Table 5:** Verification of accuracy (n = 3).

Groups	Test Value (pg/μL)	Spike Recovery (%)	Average Spike Recovery (%)
High-dose spiked group	19.726	118.33%	123.2
	21.289	127.71%	
	20.606	123.61%	
Medium-dose spiked group	1.422	85.02%	101.3
	1.627	97.34%	
	2.030	121.50%	
Low-dose spiked group	0.019	86.88%	96.1
	0.021	101.25%	
	0.021	100.22%	
Samples	0.005	-	-

**Table 6:** Verification of repeatability (n = 6).

Groups	Test Value (pg/μL)						$\bar{x}$ ± **D**	RSD (%)
Samples	0.009	0.009	0.011	0.010	0.012	0.010	0.01017 ± 0.00117	11.5
Spiked samples	1.716	1.533	1.805	1.548	1.630	1.503	1.6225 ± 0.11828	7.3

**Table 7:** Verification of intermediate precision (n = 12).

Groups	Personnel	Test Value (pg/μL)						$\bar{x}$ ± **D**	RSD (%)
Samples	A	0.009	0.009	0.011	0.010	0.012	0.010	0.01033 ± 0.00098	9.5
	B	0.011	0.010	0.012	0.010	0.010	0.010		
Spiked samples	A	1.716	1.533	1.805	1.548	1.630	1.503	1.67242 ± 0.18020	10.8
	B	1.489	1.492	1.753	2.034	1.634	1.932		

**Table 8:** Recombinant plasmid drug residual host cell RNA test results.

Batch Numbers	Sample Type	RNA Content (pg/μL)	Spike Recovery (%)	Plasmid Content (mg/ml)	Residual Host Cell RNA (μg/mg DNA)
NLJC202412001	first chromatography pool	16.8388	103.3	0.72	0.0233872
	second chromatography pool	0.0176	104.6	1.00	0.0000176
	third chromatography pool	0.0176	99.1	1.07	0.0000164
	drug substance	0.0154	77.4	2.00	0.0000077
NLJC202412002	first chromatography pool	5.4868	92.9	0.78	0.0070343
	second chromatography pool	0.0396	84.4	0.99	0.0000400
	third chromatography pool	0.0220	84.7	1.14	0.0000193
	drug substance	0.0198	105.6	2.02	0.0000098
NLJC202412003	first chromatography pool	2.4156	79.8	0.76	0.0031784
	second chromatography pool	0.0396	79.0	0.96	0.0000412
	third chromatography pool	0.0220	79.7	1.03	0.0000214
	drug substance	0.0330	95.1	2.00	0.0000165
NLJC202412004	drug substance	0.0220	111.2	2.08	0.0000106
01JC202311003	drug substance	0.0242	102.5	2.02	0.0000120
01JC202311004	drug substance	0.0726	106.8	2.03	0.0000358
01JC202312005	drug substance	0.0330	114.3	1.94	0.0000170

## Data Availability

The original contributions presented in this study are included in the article. Further inquiries can be directed to the corresponding authors.
